# Prosthodontic Rehabilitation of Hereditary Ectodermal Dysplasia in an 11-Year-Old Patient with Flexible Denture: A Case Report

**DOI:** 10.1155/2012/489769

**Published:** 2012-12-22

**Authors:** Neha Jain, Dinesh Naitam, Arti Wadkar, Anuradha Nemane, Shiva Katoch, Ashish Dewangan

**Affiliations:** ^1^Department of Prosthodontics, Manav Rachna Dental College, Faridabad 121004, India; ^2^Department of Prosthodontics, Rungta College of Dental Sciences and Research, Kohka-Kurud Road, Kurud, Bhilai 490024, India; ^3^Department of Prosthodontics, Nair Hospital Dental College, Mumbai 400008, India; ^4^Department of Prosthodontics, YMT Dental College, Navi Mumbai 410210, India; ^5^Department of Prosthodontics, D.A.V. Dental College, Solan 173212, India

## Abstract

Hereditary ectodermal dysplasia is a rare group of inherited disorders characterized by aplasia or dysplasia of two or more tissues of ectodermal origin such as hair, nails, teeth, and skin. The dental characteristics of this syndrome include anodontia or hypodontia of the primary and/or permanent teeth, hypoplastic conical teeth, and underdevelopment of the alveolar ridges. The options for a definitive treatment plan include fixed, removable or implant-supported prostheses, singly or in combination. This clinical report describes the prosthetic rehabilitation of an 11-year-old boy with hereditary ectodermal dysplasia. Maxillary flexible removable partial denture and mandibular conventional complete denture were fabricated to establish an acceptable masticatory function, speech, and esthetics for the patient.

## 1. Introduction

Ectodermal dysplasia (ED) is a large, heterogeneous group of inherited disorders that are characterized by primary defects in the development of two or more tissues derived from the embryonic ectoderm. The syndrome involves overlapping features, thereby complicating a definitive classification. The two main categories of ED are the Hidrotic (Clouston's syndrome) and Hypohidrotic (Christ-Siemens-Touraine syndrome) forms [1]. The difference between the two is in terms of sweat gland manifestations. Hidrotic ectodermal dysplasia has an autosomal dominant inheritance where sweat glands are normal which are absent in Hypohidrotic ectodermal dysplasia. The latter (HED) has an X-linked recessive inheritance and is the most common form of ED syndrome affecting men more severely and frequently [1]. The incidence of HED is about 1/100,000 [2].

Patients with HED have absent or decreased sweating because of lack of sweat glands producing extremely high fevers because their skin cannot control temperature properly. The skin is thin with light coloring. Hair may be absent or very thin. After puberty hair growth improves in some patients. The eyebrows, eyelashes, and other body hair may also be absent or sparse. Fingernails and toenails may show faulty development and be small, thick or thin, brittle, discolored, cracked, and/or ridged.

Extraoral manifestations include frontal bossing, depressed nasal bridge, protuberant lips, and hypotrichosis. The dental characteristics include anodontia or hypodontia of the primary and/or permanent teeth, hypoplastic conical teeth, and underdevelopment of the alveolar ridges [3, 4]. When teeth are missing the alveolar bone in which they are ordinarily embedded does not develop well, leading to a reduced vertical dimension and a typical aged appearance in the face [5, 6]. The deviation from normal facial growth of HED subjects tends to lessen with age when rehabilitated with functional and prosthetic appliances [7].

Factors such as patient's age, stage of growth in conjunction with the missing teeth, soft tissue defects, existence of malformed dentition, and psychological status must be considered in the treatment planning [8]. Since patients with ED have psychosocial issues due to the orofacial manifestations presenting at such young age therefore restoring appearance and function is more challenging than usual. There are multiple treatment options for this condition, but the most frequent prosthetic treatment of ED in young patients is removable prosthodontics [9]. There is enough literature on complete dentures being quite commonly fabricated for anodontia patients till a more definitive prosthesis is fabricated [10].

This clinical report explores the use of flexible denture base material for the fabrication of maxillary removable partial denture (RPD) and conventional acrylic resin for fabrication of mandibular complete denture for a paediatric patient with hypohydrotic ectodermal dysplasia.

## 2. Case Report

**Figure 1:**
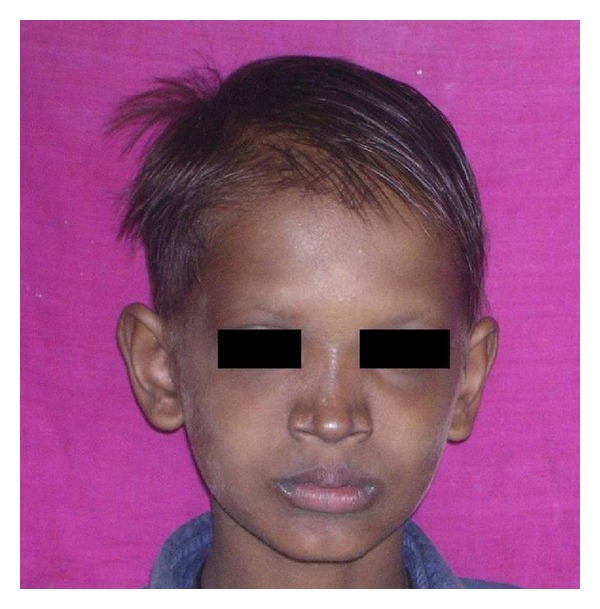
Pretreatment extraoral view.

An 11-year-old boy reported with chief complaint of inability to masticate and unesthetic appearance. He desired replacement of his missing teeth. The patient gave history of lack of sweating, dryness of skin, and raised body temperature. Extraoral examination revealed sparse hair, frontal bossing, depressed nasal bridge, prominent supra orbital ridges, sunken cheeks, hyperpigmented skin around the eyes, protuberant lips, and decreased lower facial height (Figure 1). Nails appeared normal.

**Figure 2:**
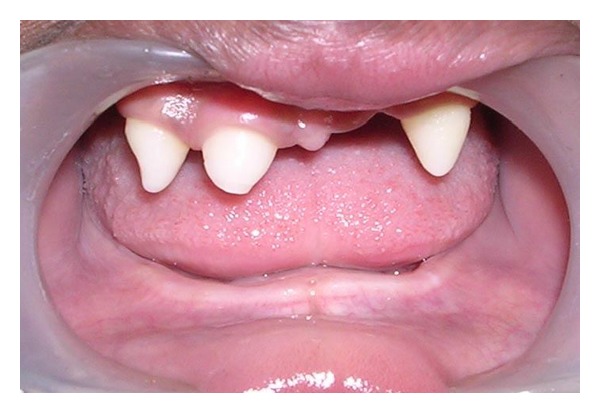
Pretreatment intraoral view.

Intraoral examination revealed absence of saliva and dry oral mucosa. Cone-shaped teeth were present in 11, 12, and 23 region with underdeveloped edentulous mandibular alveolar ridge (Figure 2).

**Figure 3:**
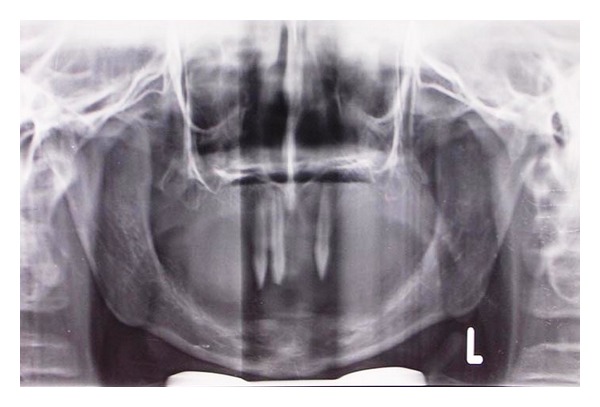
Orthopantograph-showing presence of 11, 12, and 23.

Panoramic radiograph showed presence of 11, 12, and 23 with complete root formation and there was no evidence of any impacted tooth (Figure 3).

Family history revealed similar findings in his 18-year old brother. Clinical findings and family history suggested a diagnosis of hypohydrotic ectodermal dysplasia [11].

**Figure 4:**
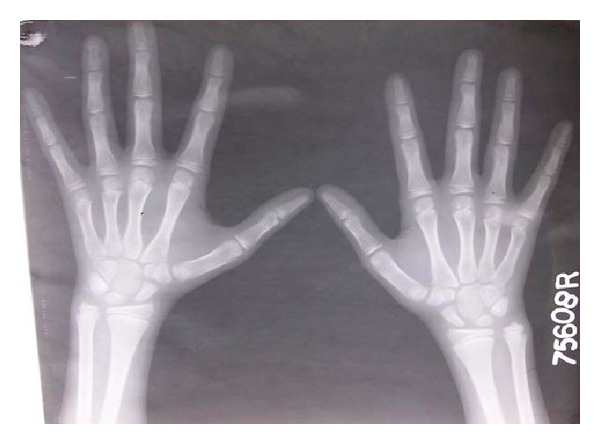
Hand-wrist radiograph for growth-spurt assessment.

Hand wrist radiograph (Figure 4) was taken and evaluated to assess the skeletal development of the patient.

After studying the skeletal maturation indicators as given by Fishman [12], it was found that no definitive prosthesis could be planned for another 6 years as the skeletal maturation was incomplete. Maxillary flexible RPD and mandibular complete denture were planned to provide immediate aesthetic results, reestablish the occlusion by replacing missing teeth and allow the patient to become familiar with removable prosthesis before delivery of the definitive prostheses [13]. Valplast flexible partial denture was planned for maxillary arch due to its favourable properties like esthetics, strength, biocompatibility, and comfort to the patient [14]. Valplast flexible denture base material uses the RetentoGrip tissue bearing technique for retention, and hence no tooth or tissue, preparation is needed [15].

**Figure 5:**
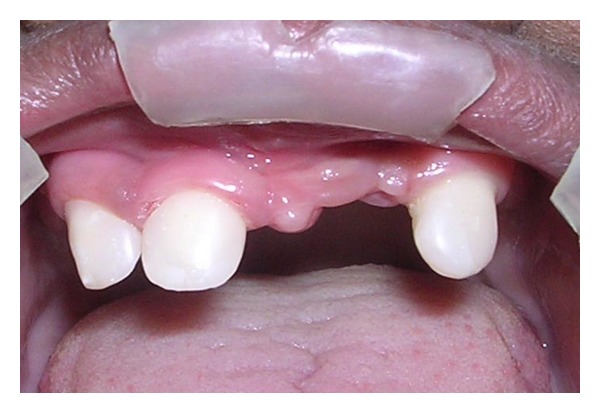
Composite restorations of 11, 12, and 23.

 Maxillary right central and lateral incisors and maxillary left canine were morphologically restored with composite resin to produce 0.25-mm undercuts [16] (Figure 5).

**Figure 6:**
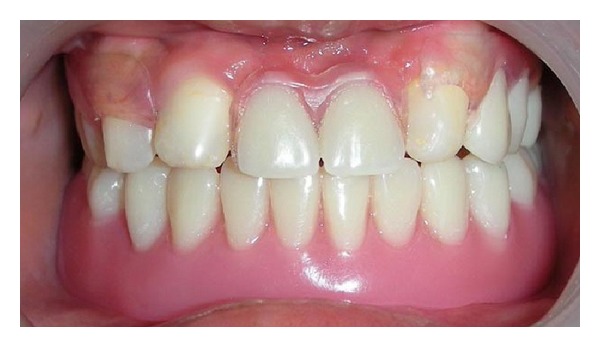
Post insertion Intraoral view.

**Figure 7:**
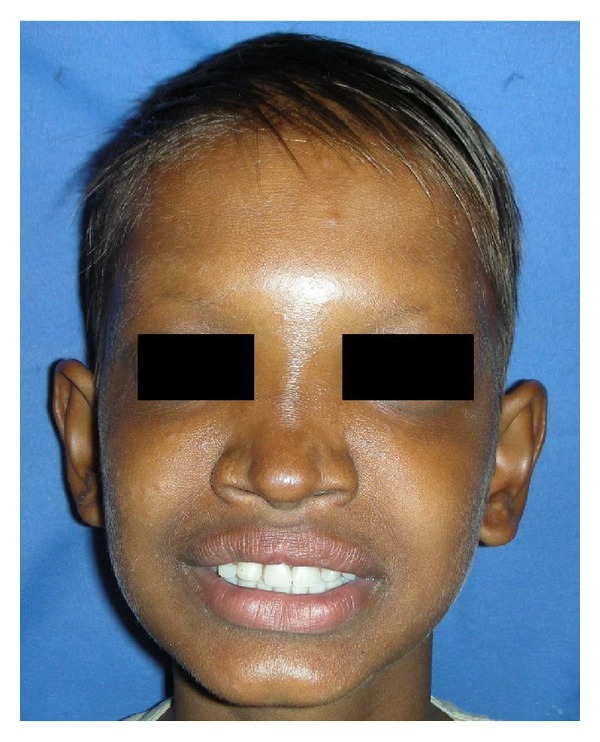
Post insertion Extraoral view.

Primary impressions were made with irreversible hydrocolloid impression material as it is comfortable and can be easily removed from undercut area. Casts were prepared with type III dental stone. Custom trays were prepared, and border moulding was done with heavy body polyvinyl siloxane material. The final impressions of the maxillary and mandibular arches were made with medium and light body type of rubber base impression material, respectively. Maxillo-mandibular relation was recorded, and the master casts were mounted on a semiadjustable articulator. The teeth were arranged according to a balanced occlusal scheme [17]. Try-in was done and after careful evaluation the maxillary prostheses were fabricated in Valplast resin by injection moulding technique, and the mandibular prosthesis was fabricated in the conventional heat cure acrylic resin. The dentures were then inserted in the patient's mouth (Figures 6 and 7).

Prior to insertion the valplast partial was immersed for a minute in warm water. The heat exposure will assure a very smooth initial insertion and allows the denture to adapt to the underlying tissue. The patient was educated about proper insertion and the removal of the prostheses and instructed about maintaining denture hygiene. Recall was done after 24 hrs to make necessary adjustments. Future visits were scheduled for 6 months to monitor bone growth and for denture relining.

## 3. Discussion

An early age prosthodontic intervention helps the child to adjust with the prosthesis and develop normal appearance, speech, mastication, and swallowing as well as temporomandibular joint function. The intraoral prosthesis can be modified during growth spurts or rapid growth periods. Apart from dental benefits, an early age intervention also provides psychosocial benefits [18].

Reduced alveolar bone height with “knife-edge” morphology and limited remaining tooth structure make prosthodontic rehabilitation a challenging task. The replacement of teeth by implants is usually restricted to patients with completed craniofacial growth and is probably best to hold off until adolescence. Implants placed in ED patients younger than 18 years have a higher risk of failure [19]. Implant insertion in children or adolescents can have several unfavourable potential effects including trauma to tooth germs and multidimensional restrictions of skeletal craniofacial growth. In this case implant therapy was not the treatment choice due to ongoing growth and development and insufficient alveolar bone support.

For young patients the use of removable partial denture (RPD) is a reversible treatment that can significantly improve functions and esthetics without jeopardizing compromised dentitions [20]. The use of metal clasps on anterior teeth may cause esthetic problems with the use of cast partial denture [21]. Recently, acetal resins have been used as an alternative tooth-coloured denture clasp material to improve esthetics [22]. The therapeutic use of thermoplastic materials like valplast has increased drastically in the late decade. This new procedure, during which a fully polymerized basic material is softened by heat (without chemical changes) and injected afterwards, has opened up a new chapter in making dentures.

Flexible dentures have got various advantages over the traditional rigid denture bases [23]. Translucency of the material picks up underlying tissue tones, making it almost impossible to detect in the mouth. No clasping is visible on tooth surfaces (when used in manufacturing of clear clasps), improving aesthetics. The material is exceptionally strong and flexible. Good biocompatibility is achieved because the material is free of monomer and metal, these being the principle causes of allergic reactions in conventional denture materials. Flexible dentures will not cause sore spots due to negative reaction to acrylic resins and will absorb small amounts of water to make the denture more soft tissue compatible. There is no requirement of tooth preparation as in case of cast partial denture.

Although dentures are poor alternatives to healthy dentition, they create conditions for the maintenance of a normal, satisfactory daily diet for the child. This is very important, considering that the establishment of lifelong dietary patterns occurs during childhood [24]. When planning dentures in these patients, care should be taken to obtain a wide distribution of occlusal load fully extending the denture base hence balanced occlusion should be the preferred occlusal scheme.

In this case the patient was very comfortable with the valplast partial denture due to its light weight and was quite satisfied with the esthetics because of absence of the metallic part usually seen in other cast partial dentures. Good retention was observed, and the parents reported a significant improvement in terms of speech and mastication.

## 4. Clinical Significance

The present clinical report demonstrated that maxillary flexible RPDs associated with direct composite restorations and mandibular complete denture are a reversible, relatively inexpensive method of treatment for ectodermal dysplasia patients especially during their growth years. The esthetic, light weight, and good retentive and fracture properties of the flexible denture base materials make them a good alternative to the conventional cast partial dentures.

## 5. Manufacturers' Details

 Irreversible Hydrocolloid-Tropicalgin, Zhermack, Italy.
 Type III dental stone-Kalabhai, Mumbai, India.
 Semiadjustable Articulator-Teledyne Hanau, Buffalo, New York, USA.
 Composite Resin-Filtek Z350, 3 M ESPE, Seefeld, Germany
 Rubber Base impression material-AFFINIS Heavy body, Medium body, Light body; Coltene Whaledent.
 Autopolymerising resin for custom trays-Pyrex; Pyrex polymer, Roorkee, India.
 Valplast Resin-Valplast Int.Corp, Long Island City, New York, U.S.A.
